# Women’s Cognition and Attitude with Eco-Friendly Menstrual Products by Consumer Lifestyle

**DOI:** 10.3390/ijerph18115534

**Published:** 2021-05-21

**Authors:** Jui-Che Tu, Ting-Yun Lo, Yi-Ting Lai

**Affiliations:** 1 Graduate School of Design, National Yunlin University of Science & Technology, Yunlin 640301, Taiwan; tujc@yuntech.edu.tw; 2 Department of Creative Design, National Yunlin University of Science & Technology, Yunlin 640301, Taiwan; a0976446672@gmail.com

**Keywords:** menstruation, eco-friendly menstrual products, lifestyle, cognition and attitude

## Abstract

With rapid technology developments and the convenient and fast pace of life in recent years, many people are using disposable products, which cause environmental and ecological damages. A variety of eco-friendly menstrual products have been launched on the market, and “menstrual pads” now have a large market share in Taiwan’s menstrual product industry. This study interviewed experts and collected questionnaires for qualitative and quantitative investigation and analysis. The results show that women have positive and open concepts regarding sustainability, as well as a good understanding of their body and are very interested in the performance and usage efficiency of products. The results also indicate that consumers purchase based on their lifestyles; most women collected enough product information before purchasing while overcoming the difficulties in dealing with the environment and learning to adapt them, and the majority of consumers attach importance to comfort, volume, and duration of blood absorption. The results suggest that the government and private enterprises should increase and improve sanitation education and sanitary facilities and guide the approach and serve as an important reference index for the promotion of eco-friendly menstrual products for environmental benefits.

## 1. Introduction

With the rapid development of science and technology in recent years, people are changing their lifestyle to mass manufacturing, mass consumption, and mass waste, which causes ecological and environmental damages. With the outbreak of COVID-19 in 2020, the importance of environmental sustainability is being recognized. The United Nations has proposed 17 goals for the sustainable development of a broad vision and called for forward-looking actions.

With the growing awareness of sustainability, eco-friendly menstrual products, such as cloth menstrual pads, menstrual cups, and menstrual underpants, have been launched on the market. According to the statistics of the Taiwan Environmental Information Center, a woman uses at least 10,000 menstrual pads in her lifetime, which has a negative impact on earth’s environments [1]. Menstrual products on the market are divided into disposable and eco-friendly products. The former includes menstrual pads and menstrual tampons, which are mainly composed of cotton, non-woven fabrics, and polymer absorbers, are non-recyclable and must be buried or incinerated. It takes 50 years for a pad to decompose, and if incinerated, its chlorinated, disinfected, and bleached pulp ingredients produce dioxins and other harmful substances that eventually enter the food cycle, which not only pollutes the environment but also endangers natural species. The same issue exists for baby diapers. Since 2014, more and more mothers have been making cloth diapers themselves to save money and protect the environment, while cloth menstrual pads, which are similar to cloth diapers, are poorly accepted among women, because they are difficult to clean and cannot fit the busy pace of modern life [2,3,4,5,6,7,8,9].

During menstruation, women use different menstrual products to absorb menstrual blood and prevent outflow. The use of menstrual products can be traced back to ancient times, when women used leaves, hay, and bark, which are easy to clean, to carry menstrual blood due to poor living conditions [10,11,12,13,14,15,16,17,18,19,20,21,22]. However, women in different countries dealt with menstrual blood in different ways, including the use of wool, folded paper, and papyrus. With the progress of social civilization, textiles have been widely used by contemporary people since their occurrence. In China, women put water-absorbing materials, such as grass and wood ash or silk and hemp into a small cloth bag and tied it around their waists with thin strings at the two ends. Such bags could be cleaned and reused, and women called them “menstrual bags”. In the 19th and early 20th centuries, most women in western countries used self-made menstrual pads that could be washed and reused repeatedly. Southalls’ Pads, invented by Franklin in 1888, were first used as trauma pads due to their excellent hemostatic performance and were the first disposable menstrual pads when they were used by nurses as disposable sanitary products; thus, they were the origins of disposable menstrual pads [23,24].

The UN especially mentions females in its fifth goal of sustainable development, which suggests that women are not only a subject of significance but they also play a key role in the process of promoting sustainable development. Women are both producers and incubators, but their productivity is hindered by social and family inequalities. In the past, women in most developing countries did not own lands and farms, but as they spent a lot of time maintaining lands to support their families, they learned more about soil, plants, and trees, thus, according to the theories of eco-feminists, they had a better understanding of the environment and could manage the environment in a more sustainable way than men [25]. The results of relevant studies have shown that countries with more women engaging in the government or market are less likely to have corruption issues, and from the moral point of view, women protect the environment with a sort of natural moral concept formed in a non-corrupt manner; therefore, they also protect natural resources [26]. Other statistics also show that 80% of household shopping is done by women; thus, purchasing related to the environment should be in the control of women [27]. The unequal playing field between men and women has enormous economic costs, as it impedes the increase in productivity. The world is responsible for realizing equal actions and exchange between men and women in the next century.

Among women’s attitudes regarding eco-friendly menstrual products, cloth menstrual pads and menstrual cups are the main trends, followed by menstrual underpants, while menstrual pads and tampons account for the market majority of menstrual products. While tampons are superior to menstrual pads in all aspects, women prefer the latter, as the former are invasive, and people are afraid of pain and have a misunderstanding of a “damaged hymen” [28,29,30,31,32,33]. In addition, women’s refusal to use invasive products is also the result of the irrational interpretation of female organs by society. There is also discrimination against women using menstrual cups all over online media. They put forward the strict criteria to determine “whether a woman is a virgin” and impose the pressure of public opinions on women, which affects their purchase of menstrual products [34,35].

Modern people live a busy life and prefer fast and convenient disposable products; thus, the invention of disposable menstrual pads met the pursuit of modern women for convenience [36]. In recent years, manufacturers in the market have used new technologies and materials in menstrual pads to make the products lighter and thinner, in order to minimize the discomfort of women during menstruation and improve their willingness to use menstrual pads [37]. With advances in technology, innovative atypical menstrual products are developed, which make menstrual products on the market more diversified and cause women to promote menstrual pads, menstrual cups, and menstrual underpants. With increasing discussions of women’s autonomous right of their bodies on the Internet and in society, new forms of menstrual products are making a comeback [38].

**Figure 1:**
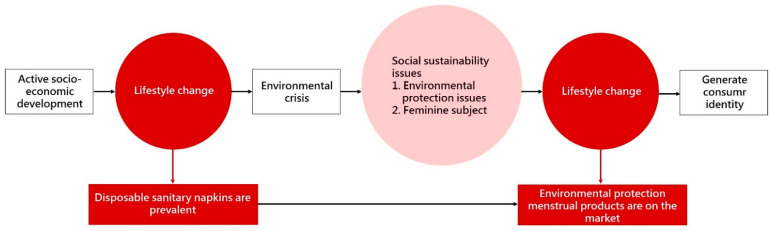
Research Correlation Graph.

Modern people are accustomed to lives of convenience, pursuing material growth and holding onto the idea that science and technology will solve all their problems. Therefore, disposable products have flooded our lives. People are used to disposable products, including disposable sanitary napkins, which are necessary menstrual products in a woman’s life. Various harmful substances can be generated when recycling sanitary products and are likely to eventually enter the food chain. This will not only cause environmental pollution, but it also endangers natural species and their habitats. In view of the seriousness of the above problems, reusable environmentally friendly menstrual products are an alternative to replace women’s physiological needs and reduce the environmental burden. This study intends to explore consumers’ cognition and attitude toward environmentally friendly menstrual products. According to the current living conditions of female groups, data analysis and feedback collation were carried out, including personal internal factors and social external factors, in order to promote the reduction of disposable products and the promotion of the use of environmentally friendly menstrual products, as shown in Figure 1. The purpose of this study is as follows:

1. This paper analyzes the development of menstrual products by literature and compares disposable and reusable variations, so as to understand the representative significance of different menstrual products at the demand level and environmental protection level.
2. Through expert interviews, this paper aims to understand the development status and future trends of the menstrual products market and analyze consumers’ needs at different levels from a marketing perspective.
3. Through questionnaire surveys, this paper analyzes consumers’ cognitive concepts on menstruation and sustainability issues and compares their motives and doubts about the use of environmentally friendly menstrual products.
4. To explore the factors that consumers consider when purchasing environmentally friendly menstrual products and understand the consumption identity and resistance of menstrual products.
5. To summarize menstrual products for simulation analysis, extract ethnic lifestyle patterns and influencing aspects of using products, and provide a reference basis and indicators for future sustainable issues in environmentally friendly menstrual products planning.

## 2. Materials and Methods

### 2.1. Research Process

**Figure 2:**
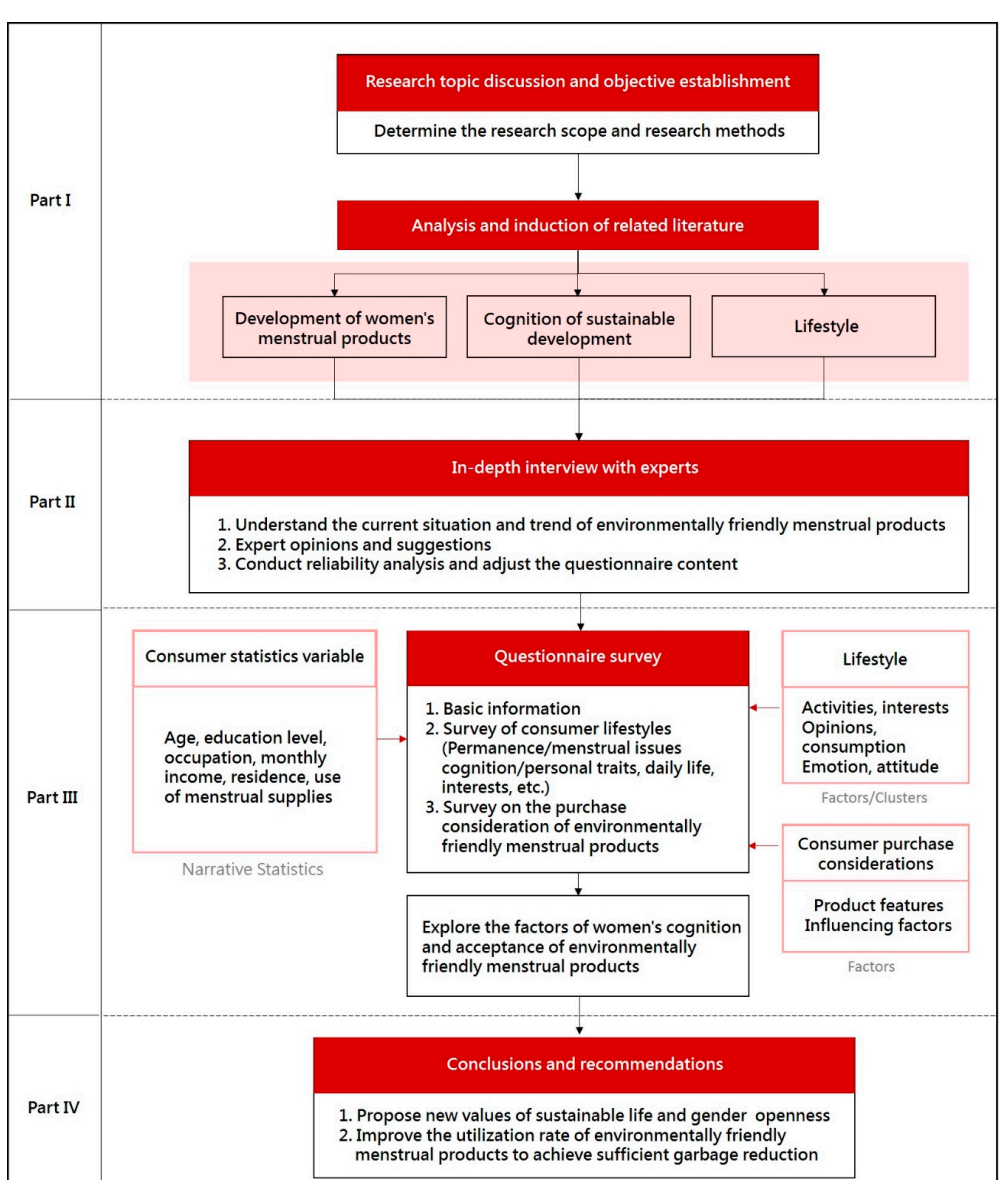
Research architecture diagram.

This study was first carried out qualitatively and then quantitatively. In phase 1, a literature review was conducted to collect the information of menstrual products, sustainable development, and lifestyle, and the outlines and key points were summarized [39,40]. In phase 2, the interview outline was determined according to the literature review, and experts in different fields were interviewed in a semi-structured manner. The feasibility of the questionnaire survey was also studied in this phase. In phase 3, provisional questionnaires were designed. Questionnaires in this study were used to learn about the cognition and attitude of eco-friendly menstrual products by the women of the different groups. Finally, IBM SPSS 22 software (International Business Machines Corporation, New York Armonk, USA) was used to analyze the questionnaire results, as shown in Figure 2.

### 2.2. Research Targets

Different types of menstrual products available on the market were analyzed for strengths and weaknesses, as shown in Table 1, in order to learn the main functions of the products [1]. Different consumer groups have different demands in use, and most of them mainly require the prevention of outflow, comfort, and affordable sales price. Such different demands of consumer groups lead to diversified functions of menstrual products on the market [2,3,4,7].

This study conducted expert interviews and a questionnaire investigation. Provisional questionnaires were designed and then adjusted into the formal questionnaires containing 80 questions according to experts’ advice. A total of 448 online questionnaires were collected, and 8 invalid questionnaires were excluded; thus, 440 questionnaires were included in this study to learn about the cognition and attitude of eco-friendly menstrual products by the women of the different groups. The subjects of this study were women aged from 18 to 50 years old. Since the average age of women’s first menstruation is 12 years old when they had no sufficient knowledge of menstrual products and lacked independent judgment and economic capability, and the average age of their menolipsis is between 48 to 52 years old, female consumers aged from 18 to 50 years old dealing with menstruation were included as subjects. In order to analyze the differences in cognition and attitude between different groups, the subjects were grouped into three age ranges to identify the factors related to women’s recognition of eco-friendly menstrual products. Finally, IBM SPSS 22 software was used to analyze the questionnaire results from the aspects of reliability and narrative statistics, factors, and clusters, and the differences were compared and summarized in order to identify the influencing relationships and constructs between the different lifestyles of the different subjects.

### 2.3. Research Subjects

This study was carried out in two phases. In phase 1, experts, including one representative academic researcher on gender issues in Taiwan and two practitioners of eco-friendly menstrual products, were interviewed in a semi-structured manner, and their information is shown in Table 2. The interviews were intended to learn about the opinions of consumers on sustainable environmental protection, as well as consumers’ feedback on different menstrual products on the market, in order to understand the market operation of existing eco-friendly menstrual products and the key factors for products to focus on the revolution of menstruation [28,46,47]. The interviews were recorded, and key points were identified, analyzed, and organized, in order to fully understand the interview information to provide a reference for future studies.

In phase 2, questionnaires were sent to and collected from female consumers with independent economic capability who could buy their menstrual products at will to learn about the opinions of women of different age groups. As summarized and analyzed, the factors affecting women’s purchase of eco-friendly menstrual products were identified, and their correlation was analyzed for studies on menstrual products [48].

## 3. Results

### 3.1. Analysis of Expert Interviews

In this study, the interview outline was determined based on the key points summarized from the literature review, and experts in relevant fields were interviewed. In order to acquire in-depth feedback from experts, the interviews were guided by questions about different constructs and were not limited to the preset questions [39,40].

Three experts provided advice in different fields in this study. Associate Professor Lin talked about consumers’ current use of cloth menstrual pads, who such consumers are, and the price of environmentally friendly menstrual products, and how academic promotion reflects the difference in the cognition of menstruation among the young generation. Manager Shi, a former project manager of the “menstrual cup” and current developer of menstrual underpants, talked about the marketing of atypical menstrual products and offered information regarding the current development trends of menstrual cups and menstrual underpants, how consumers’ lifestyles affect the usage habits of menstrual products, and the conditions for the promotion of relevant products. Designer Chen, a designer of menstrual cups and menstrual underpants, talked about the appearance, functions, and structural differences of different types of menstrual cups, as well as users’ usage habits and concerns about comfort during the development of the products [1,28,29].

The interviews mainly covered three aspects, namely (1) the impact of sustainability and consumers’ lifestyles, (2) the current development of eco-friendly menstrual products, and (3) the characteristics and elements of eco-friendly menstrual products, and the three main categories of this study were determined according to the results. Then, several sub-categories and open decoding were converged in order, and the key elements of context decoding were extracted, summarized, and integrated according to six constructs, as shown in Table 3.

As shown above, the differences in the lifestyles between individuals had special importance in the analysis of the user groups. Both the development of eco-friendly menstrual products affected by lifestyle and the difference between individuals in the use of eco-friendly menstrual products were related to social conduct, age, stratum, occupation, and personal traits. According to the focus of the above-mentioned expert interviews, a follow-up questionnaire survey on consumers was drawn up with a 5-point Likert scale, as shown in Table 4. By analyzing the lifestyle and purchasing considerations of different consumer groups, this paper explores the cognition and attitude of consumer groups and the acceptance factors of other non-environmental menstrual products users [1,46].

### 3.2. Analysis of Consumers’ Lifestyles

The AIO scale was used in this questionnaire investigation, and IBM SPSS 22 software was used for quantitative analysis, in order to investigate consumers’ opinions regarding sustainability and menstruation and examine the constructs, including daily lifestyle, personal traits, and interest in and purchase of eco-friendly menstrual products, which was intended to define the correlation between different groups regarding their willingness to purchase eco-friendly menstrual products [49,50].

(1) Analysis of consumers’ use of menstrual products
  In this study, eco-friendly menstrual products were divided into cloth menstrual pads, menstrual cups, and menstrual underpants. Considering that many consumers might be using two or more types of these menstrual products at the same time during menstruation due to product diversity in the market [46,51], the combinations of different types of menstrual products were set as the investigation items.
  As shown in Table 5, 60.7% of the subjects used menstrual pads, which still account for the majority in Taiwan, while fewer subjects used tampons; however, the percentage was significantly higher than before, indicating that invasive products were less concerned by women. Among the eco-friendly menstrual products, 48.9% of the subjects used menstrual cups, accounting for the majority, while fewer subjects used cloth menstrual pads, with the percentage similar to the previous percentage. The percentage of menstrual underpants users was the lowest, at only 13%, as the products were not popular and were emerging atypical menstrual products.
(2) Analysis of consumers’ attitude on menstrual products
  1. Concerns about the use of menstrual pads
    When using menstrual pads, 84.1% of the subjects were concerned about discomfort, 80.5% were concerned about their disposal, and 65.7% were concerned about the harm to the environment, which indicates that the public attach high importance to sustainability. In addition, 63% of the subjects were concerned about the outflow of blood, and 52.7% were concerned about vaginal infections, which is due to the characteristics of the products. Some subjects were also concerned about the cost, as they had to spend more on them due to heavy blood flow during menstruation [26,27,52].
  2. Preference for the type of eco-friendly menstrual products
    In the investigation of the willingness to use eco-friendly menstrual products, the effective percentage of the subjects who were willing to use the products was as high as 89.5%, which suggests that the current society is interested in pursuing sustainable ecological friendliness and requires sustainable eco-friendly menstrual products.
    By excluding those not willing to use eco-friendly menstrual products, a total of 394 subjects were investigated to determine their preference for the type of eco-friendly menstrual products. According to the experts interviewed in this study, most consumers preferred menstrual cups, accounting for 61.9%, while 26.1% of them preferred cloth menstrual pads, because it was troublesome to clean the latter and users preferred convenience and cleanliness. In addition, only 11.9% of the subjects preferred menstrual cups, as they are new to the market, and little is known about them.
  3. Motivation for the use of eco-friendly menstrual products
    Combinations of different types of eco-friendly menstrual products were included as options in this study to learn about the main motivations among consumers regarding the use of eco-friendly menstrual products. Most subjects, accounting for 75.9%, used them to respond to the call for environmental protection, while 67.3% used the products for higher comfort and 75.3% used them for convenience. These results are in line with the analysis results of the concerns about the above-mentioned use of menstrual pads, indicating that modern women are also pursuing high quality of life. Moreover, many subjects choose eco-friendly menstrual products due to their inherent extra strengths, such as the convenience of menstrual cups for exercise and water sports, and the different patterns of cloth menstrual pads. A few subjects choose these products to save money [2,52].
  4. Concerns regarding the use of eco-friendly menstrual products
    Subjects of the same group had almost the same concerns regarding the use of eco-friendly menstrual products, which reflects their different needs in life. Those not using eco-friendly menstrual products stated their concern about infections and pain caused by invasive products [1,2], which indicates that the current development of menstrual cups is still limited to a certain group of consumers, while those who had a correct understanding of physiology were using invasive products properly after learning and were not likely to have infections resulting from inadequate disinfection [2,47]. This analysis result is in line with the point of view of Expert C, meaning when a product is well developed and its user group expands, some use it before having a good understanding of sanitation, which results in improper use and infections. Some users also raised the concern that it is inconvenient to use eco-friendly menstrual products in the current environment, as they need to be cleaned and few facilities are available. The government and private enterprises should make efforts to improve this situation in the future [53].
(3) Analysis of consumers’ cognition and behavior
  The 5-point Likert scale was used to measure the subjects’ cognition of sustainability and menstruation. Cognitive and behavioral decisions are derived from the theory of planned behavior, which affects the constructs, including behavioral beliefs, behavioral attitudes, normative beliefs, subjective beliefs, perceived behavioral control, and behavioral intentions [54].
  The analysis of consumers’ cognition of sustainability is shown in Table 6. Although some subjects did not agree to sustainability, the subjects agreed or strongly agreed to sustainability on the whole, indicating that the public has a high sense of identity regarding sustainability and would act to protect the environment.

In the investigation of menstruation, all items, except Item 10 (I think menstruation is a normal physiological phenomenon of women) and Item 13 (Women should have the right to take menstrual leave from school or work), were negative statements, as shown in Table 7. Most subjects agreed with positive statements and disagreed with negative statements, indicating that most modern women had different cognitions and behaviors of menstruation compared to traditional ones. It should be noted that most subjects agreed to the negative Item 4 (I think menstruation brings inconvenience to life), as women deal with menstruation in different ways because of their physiological structure, which is different from men, and some women had pain during menstruation [34]. In the future, social forces should attach importance to such issues when building women-friendly facilities [53].

### 3.3. Analysis of Consumers’ Factors and Clusters

This section analyzes the differences in consumers’ lifestyles. Factor analysis was used to simplify the items regarding consumers’ lifestyles and to extract the factors of types of lifestyles, in order to learn the characteristics of the different lifestyles of consumers. Exploratory factor analysis (EFA) was used, and principal component analysis and varimax performed orthogonal rotation to extract the main factors.

Factor analysis was carried out after reliability testing. The principal component analysis method was used to extract the main factors with eigenvalues greater than 1 as the screening condition. A total of four common factors with eigenvalues greater than 1 were extracted, and the accumulative explanatory variation was 49.228%, which met the screening criteria, as shown in Table 8.

Varimax was then used to perform orthogonal rotation of the factors, and after some items were removed according to the removal criteria, the items were simplified into four main factors, as shown in Table 9.

This study named four factors according to their meanings, “new information seeking”, “innovation trend”, “strict budgeting”, and “easy-going and conservative”.

Cluster analysis was conducted according to the items in the AIO scale. The AIO scale is defined as:

1. A (Activity): It is a specific action, such as media viewing, shopping, etc. Although these actions are common, the reasons for these actions are rarely directly measured.
2. I (Interest): The degree of interest in a thing or subject, and continuous and special attention to it.
3. O (Opinion): It is an individual’s oral or written reaction to a thing, that is, a person’s explanation, expectation, and evaluation of the thing.

Consumers were divided into three clusters. Consumers were defined by three clusters, and the correlation between the clusters was analyzed using the K-means cluster analysis method. As shown in Table 10, the corresponding values show that Cluster 1 was positively correlated with Factor 1 (new information seeking) and Factor 2 (innovation trend), and negatively correlated with Factor 3 (strict budgeting) and Factor 4 (easy-going and conservative), which indicates that people in Cluster 1 prefer to follow trends, know how to get new information using Internet tools, and are willing to spend time on things that they think are worthwhile. Cluster 2 was positively correlated with all the factors, indicating that the lifestyle of the people in Cluster 2 featured all four factors, meaning they proactively sought new information and paid attention to trends but thought carefully before purchasing due to economic restrictions. There were many people in Cluster 2. Cluster 3 was positively correlated with Factor 4 (easy-going and conservative), and negatively correlated with other factors, which indicates that people in Cluster 3 were living a conservative and stable lifestyle, their thoughts and actions were subject to the surrounding environment and policies, and they usually failed to make independent judgments. People in Cluster 3 accounted for most of the total. Based on the summary and description of all the above characteristics, as well as the key points in expert interviews, the three clusters were named: Cluster 1 is people seeking new information and new trends (aged from 18 to 28 years old and from 29 to 39 years old); Cluster 2 is people subject to economic restrictions and wanting to try something new (aged from 18 to 28 years old); and Cluster 3 is people pursuing a conservative and stable life (aged from 29 to 39 years old and from 40 to 50 years old).

### 3.4. Analysis of Consumers’ Considerations in Purchasing Based on Lifestyle

This section investigated the considerations of consumer clusters with different lifestyles when purchasing eco-friendly menstrual products, including the purchase channel of products, the volume of obtained product information, users’ feedback, brand concept, the appearance product designs, accessories, the impact of social conduct, and the properties of the products, in order to determine whether there were significant differences between the clusters, and the analysis results were summarized.

Factor analysis was carried out after reliability testing. The principal component analysis method was used to extract the main factors with eigenvalues greater than 1 as the screening condition. A total of eight common factors with eigenvalues greater than 1 were extracted, and the accumulative explanatory variation was 65.006%, which met the screening criteria, as shown in Table 11.

Varimax was then used to perform orthogonal rotation of the factors, and after some items were removed according to the removal criteria, the items were simplified into eight main factors, as shown in Table 12.

Based on the results of factor analysis, the factors obtained from the questionnaire investigation of this study were named according to the meanings of corresponding items as “use”, “product performance”, “market information”, “product design”, “market characteristics”, “benefit of use”, “market environment”, and “product value”.

Through the analysis of the characteristics of the above factors, we can know that due to the broad promotion of sustainability in recent years, women consumers are affected by the cognition of environmental protection when dealing with menstruation, and some women have begun to use eco-friendly menstrual products [37,47,55]. While the results of this study show that disposal menstrual pads are still being used by the majority, the number of women using atypical menstrual products, such as cloth menstrual pads, menstrual cups, and menstrual underpants, is increasing, and consumers were willing to try such products and experience their benefits. Atypical eco-friendly menstrual products are the future trend for the development of the market [36,46].

## 4. Discussion

Regarding consumers’ cognition and willingness to use eco-friendly menstrual products, interviews with experts showed that people have a certain understanding of environmental protection, which is the main factor affecting social conduct, and women hold positive and open opinions about menstruation and their body. The results of the questionnaire analysis support experts’ conclusions regarding products available in the market. According to the analysis tables of women’s concerns about the use of menstrual pads and their motivations for using eco-friendly menstrual products, eco-friendliness was the greatest concern among consumers, and according to the investigation and analysis of consumers’ cognition of sustainability and menstruation, all the subjects had positive cognition, and this result showed little difference between the different clusters regarding willingness to use eco-friendly menstrual products. In conclusion, modern women have learned about eco-friendly menstrual products from the Internet, and their opinions of eco-friendly menstrual products were based on positive and open cognition of the body, as well as the sense of identity to environmental protection, and they were willing to use relevant products [1,46]. However, regarding their concerns about the use of such products, women decided to purchase eco-friendly menstrual products based on their needs in life; thus, in order to meet the needs of different female groups during menstruation and improve their willingness to purchase and use the products, the products should be promoted according to consumers’ lifestyles [36,37,47].

Female groups with different lifestyles had different considerations in purchasing eco-friendly menstrual products. The qualitative results of expert interviews and the quantitative results of the questionnaire investigation focused on lifestyles and divided female consumers into those seeking new information and trends, those subject to economic restrictions and wanting to try something new, and those pursuing a conservative and stable life. Those seeking new information and trends were curious about trends, paid close attention to new information, had a basic sense of identity with eco-friendly menstrual products, attached importance to the value attached to the products, including the eco-friendly characteristics and concept of the products, and broke the established impression of feeling discomfort during menstruation. As they valued the strength of eco-friendly menstrual products to solve the inadaptability of menstrual pads, the use of the products did not stop them from purchasing the products. Those subject to economic restriction and wanting to try something new valued the added value of products and attached importance to the availability of product information to avoid wasting money, meaning they would be more willing to purchase an emerging eco-friendly menstrual product when adequate information is available. Moreover, the inherent strengths of the products, such as money-saving, convenience, the ability to be combined with other menstrual products, and the ease of observing menstrual blood, were also the considerations to purchase eco-friendly menstrual products. Those pursuing a conservative and stable life preferred to maintain their current lifestyle and did not pursue unfamiliar popular things; thus, they had more considerations, such as product performance and added value, when purchasing menstrual products. Compared with other groups, while they thought comprehensively, they attached less importance to the use of the products. The results of the expert interviews indicated that female consumers were not overly concerned with inconvenience when dealing with and learning about eco-friendly menstrual products or their inadaptability to invasive products, which is in line with the aforesaid argument that “women’s opinions on eco-friendly menstrual products were based on positive and open cognition of the body”, meaning there is positive feedback on the promotion of eco-friendly menstrual products.

Regarding the importance factors of eco-friendly menstrual products for consumers, the analysis of concerns about the “use of menstrual pads” and the “motivations to use eco-friendly menstrual products” analysis results show that consumers attach the highest importance to “eco-friendliness” and “comfort” of products, which is in line with the results of expert interviews. When purchasing menstrual products, consumers pursue comfortable products.

The questionnaire results of consumers’ concerns about the use of eco-friendly menstrual products were evenly distributed, which made it impossible to analyze the distinction between significant factors; therefore, the analysis results of expert interviews should be highlighted. All three experts mentioned that society is not friendly to users of eco-friendly menstrual products, and it is more difficult to adapt to menstrual cups because there are high initial costs in finding the most suitable type of menstrual cup, and their use requires the collection of comprehensive and complex information [4]. Users can use eco-friendly menstrual products properly only when they have a good understanding of the physiology [31].

### Limitations and Recommendations

The eco-friendly menstrual products referred to in this study are made of materials that can be reused after cleaning and disinfection and mainly include the cloth menstrual pads, menstrual cups, and menstrual underpants popular in the market. Women reach menarche between 12 and 18 years old and menolipsis between 48 and 52 years old. Considering that subjects have to be economically independent, in order to maximize the average age of menolipsis of the subjects, and comprehensively learn about the opinions of young, adult, and middle-aged women with different social and cultural backgrounds, women aged from 18 to 50 years old were included as subjects in this study. The questionnaire used in this study was a self-compiled scale, and the understanding of it by subjects might be affected by the expression of its contents; thus, there may be deviations in the feedback. This study investigated only a limited scope of groups, and the subjects were all Taiwanese; thus, the results of the questionnaire investigation cannot be applied to groups in other countries.

Relevant brands in the market have explicitly expressed their philosophies on the products’ added value; thus, the first step is to disclose more information of the products in the market, which is also an aspect that needs improvement in the future. Adequate feedback could further reduce consumers’ concerns regarding emerging menstrual products and improve the usage rate of such products [2,3]. While consumers attach higher importance to the performance and benefits of products, rather than their weaknesses in use, they have to overcome the social restrictions in use, such as the unfriendly environment and lack of facilities. Facilities in publicly accessible toilets, as well as in private enterprises, should be improved to provide users of eco-friendly menstrual products with a friendly environment to deal with the needs of menstruation [53]. Moreover, society does not provide adequate education for physiology and sanitation. Although the results of this study show that most women had positive and open cognition of the body, they are still at risk of infection due to improper knowledge of the body and inadequate preparation for when the development of menstrual products reaches its peak in the future [2,3]. Most sanitation education and online media still take disposable menstrual pads as their main samples, which limits the understanding of menstrual products among the majority of women to the well-known menstrual pads. Therefore, it is necessary to offer adequate education on health and sanitation to prepare underage girls for menarche [32,33]. As there are few legal distributors of menstrual cups in Taiwan and the types are not diversified enough, this results in difficulties for consumers to find the type of cup that suit their bodies [2,3,6]. Therefore, we recommend that the government or private organizations develop solutions to the cost issue and launch more brands and models on the market to allow women to learn about their physical structure and female consumers to buy the most suitable menstrual cups at the lowest price [27,38,52]. Compared with disposable menstrual pads, on average, eco-friendly menstrual products are cheaper if used for the long term, which is an economic motivation for their use and one of the conditions to promote them, and can provide a reference basis and serve as an indicator for the future market of menstrual products.

## 5. Conclusions

This study investigated the impact of consumers’ lifestyle on their cognition and attitude regarding eco-friendly menstrual products and focused on the factors affecting the cognition and motivation to purchase by exploring the differences of the considerations of different groups to purchase eco-friendly menstrual products. According to the analysis results of consumers’ cognition of sustainability and menstruation, as well as the attitudes toward different eco-friendly menstrual products, this study conducted clustering based on lifestyles.

In the future, women should be provided with more education on the correct knowledge of the body and use of menstrual products, in order to reduce the infection rate due to inadequate knowledge, and underage girls should be provided with more education on sanitation and physiology. Moreover, the friendliness of the social environment and facilities in public toilets should be improved, and the government and private enterprises should make joint efforts to formulate relevant regulations to reduce costs, which would allow consumers to buy the most suitable menstrual cups at the lowest price and women to enjoy the full autonomous rights of their bodies. The analysis results of this study provide a reference basis for persons and promoters engaging in relevant fields, make important contributions to the menstrual product market, and provide an approach for relevant studies in the future.

## Figures and Tables

**Table 1:** Strengths and weaknesses of menstrual products on the market.

Type	Strengths	Weaknesses	Market Price
Menstrual pads [41]	▪ Discarded after used, clean ▪ Easy to use and change	▪ Thick, uncomfortable to wear ▪ Outflow of blood if not timely changed ▪ Causes too much waste and odors, and attracts mosquitoes ▪ Single use, composed of materials that do not easily decompose, not eco-friendly ▪ Costly	USD 0.2/piece
Tampons [42]	▪ Discarded after used, clean ▪ Cause no feeling when worn, comfortable ▪ No outflow of blood	▪ Easily forgotten when worn because they cause no feeling, resulting in infection ▪ Single use, composed of materials that do not easily decompose, not eco-friendly ▪ Costly	USD 0.4/piece
Cloth menstrual pads [43]	▪ Can be reused, cause no harm to the environment ▪ Comfortable, breathable ▪ Easy to observe the menstrual blood when being cleaned	▪ Takes time to clean, which causes inconvenience and sanitary issues ▪ Outflow of blood if not timely changed	USD 10/piece
Menstrual cups [44]	▪ Can be reused, cause no harm to the environment ▪ Cause no feeling when worn, comfortable ▪ No outflow of blood ▪ Easy to observe the menstrual blood when being cleaned	▪ Takes time to clean, which causes inconvenience and sanitary issues ▪ Easily forgotten when worn because they cause no feeling, resulting in infection, but less likely than tampons ▪ To be learned and adapted to	USD 20/piece
Menstrual underpants [45]	▪ Can be reused, cause no harm to the environment ▪ Cause no feeling when worn, comfortable ▪ No outflow of blood ▪ Requires no change of the usage habit	▪ Takes time to clean, which causes inconvenience and sanitary issues ▪ Requires supplementary sanitary products in the event of a greater amount of menstrual blood	USD 20/piece

**Table 2:** Information of interviews.

Interviewee Number	Organization	Title	Relevant Experience/Research Field	Years of Service	Date/Place of Interview
A	National Dong Hwa University Department of Taiwan and Regional Studies	Associate Professor	▪ Research field: Culture and social geography, ghost geography, Taiwan regional culture, urban geography ▪ Promoter of cloth menstrual pads in Taiwan ▪ Introduced the issue of cloth menstrual pads in the second-hand cloth course	10 year	20 March 2020 Video interview
B	Artemis Medical Device Limited	Manager	▪ Founder of “Good Moon Mood”, an organization for menstrual underpants in Taiwan ▪ One of the promoters of the Menstrual Cup Funding Program in Taiwan ▪ Former project manager of “menstrual cups” in Taiwan ▪ One of the developers of menstrual underpants in Taiwan ▪ Published a research paper on Taiwan’s online tampon community	5 year	9 April 2020 Cafe
C	Artemis Medical Device Limited	Designer	▪ Co-founder of “Good Moon Mood”, an organization for menstrual underpants in Taiwan ▪ Product designer of “menstrual cups” in Taiwan ▪ One of the developers of menstrual underpants in Taiwan	5 year	9 April 2020 Cafe

**Table 3:** Analysis and summary of interviews information.

Construct of Analysis	Description
Change of lifestyle in the context of universal sustainability	Policies in Taiwan that promote use habits Life-affecting technologies Consciousness and behavior affected by people in society Influential social conducts
Factors promoting green consumption	Sense of crisis and reflection that promote eco-friendly awareness Influential social trends (external environment, online communities) Personal traits Better quality of life
Correlation between development of eco-friendly menstrual products and lifestyle	Society calls for eco-friendliness in addition to convenience and cleanliness Society calls for the autonomous selection of products in addition to the autonomous right of the body The understanding of menstruation turns from negative to positive Diversified products meet the needs of people of different lifestyles
Differences in the use of eco-friendly menstrual products between different groups	Differences in education and stratum Difference in demand between different occupations Difference in focus between different age groups Different willingness to use between people with different personal traits Positively correlated with the focus on environmental protection Nature of eco-friendly menstrual products
Current development of the menstrual product market in Taiwan	Disposable menstrual pads account for the majority There are diversified products Eco-friendly menstrual products are attracting more attention
Conditions for promotion of eco-friendly menstrual products	Attractive product design Good sanitary education More diversified products in the market Better environment and facilities for using the products Lower cost of menstrual cups

**Table 4:** Questionnaire Survey.

I. Consumer Lifestyle	
[Part I] Please describe your views on the issue of sustainability and your usual actions	
1. I attach importance to environmental protection and worry about the destruction of nature.	7. When shopping, I will pay special attention to products with environmental certification.
2. When buying goods, I will give priority to environmental protection.	8. I will learn the information of environmentally friendly products through the Internet.
3. I will bring my own environmentally friendly bags, chopsticks and cups, and try not to use disposable goods.	9. I attach importance to ecological conservation and care for the survival rights of other species.
4. I am willing to buy green goods, even if the price is more expensive.	10. I think environmental protection is more important than convenience.
5. I will reduce the production of garbage and implement garbage classification and recycling.	11. I support products that are more sustainable and environmentally friendly.
6. I don’t think the sanitary napkins used by women during menstruation are environmentally friendly.	12. I usually pay attention to issues related to environmental protection and sustainability.
[Part II] Please describe your views on menstrual issues and your usual actions	
1. I’m afraid the hymen will rupture.	9. I usually use pronouns to refer to menstruation.
2. I think invasive menstrual products will hurt the hymen.	10. I think menstruation is only a normal physiological phenomenon for women.
3. I think the deep meaning of menstrual blood is dirty and filthy.	11. I support gender equality and disagree with the traditional idea that men and women are different.
4. I think menstruation is an inconvenience to life.	12. I think menstruation is an important cycle for reproduction of life and should be paid attention to.
5. When menstruating, you should not enter the temple to worship.	13. Women have the right to take physiological leave at school or in the workplace.
6. I don’t think it’s convenient to exercise when I have menstruation.	14. In menstrual processing and self-care, I tend to hide and be silent.
7. When I have my period, I feel ashamed and find it hard to talk about.	15. 1I’m afraid of touching private parts directly.
8. I think it is shameful for others to see menstrual products, and I will especially put these products in small bags.	
[Part III] Understand your life style through descriptions of other daily life, personal characteristics, interests and opinions	
1. I like novel and fashionable things.	13. When shopping, I will focus on calculating the total cost instead of just looking at the price of goods.
2. I love some dangerous and exciting activities.	14. I think as long as the nature of the goods meets the demand, it doesn’t matter if the price is higher.
3. I do things very subjectively and don’t care what others think.	15. I think the goods used can represent a person’s concern for the things around them.
4. I prefer to do it myself rather than rely on others.	16. Users’ feedback will affect my purchase intention.
5. I pay attention to the practicality of products.	17. Innovative and unique goods are of great appeal to me.
6. I will collect product-related comments and feedback through associated networks.	18. I like a job that is challenging and changeable.
7. Vibrant, healthy and natural products can attract me more.	19. I pursue a high-quality lifestyle.
8. When shopping, I will accept or listen to the opinions of my family and friends.	20. I usually live a fast-paced and busy life.
9. I’m always learning about new knowledge and technology.	21. In the group, I am an easygoing person with less opinions.
10. I have a high acceptance of new things.	22. The norms of policies affect my consumption habits.
11. Before shopping, I will do research on the Internet to check the evaluation and price of netizens.	23. I usually pay attention to the changes of social trends.
12. When shopping, price is my first consideration.	
**II. Consumer Purchase Considerations**	
[Part I] The following are various factors that will affect your purchase of environmentally friendly menstrual products. Please fill in and answer according to your emphasis on them	
1. Convenience of product purchase.	6. Visual packaging of products.
2. Evaluation on the Internet or between relatives and friends.	7. Appearance modeling and color matching of products.
3. Feedback after personal experience of product users.	8. Parts of the product (Storage cloth bags, disinfection storage boxes, etc.).
4. Amount of information available for reference.	9. The government’s public power promotes waste reduction.
5. Brand’s main image and concept.	10. Influenced by social atmosphere.
[Part II] The following are the different characteristics of each environmentally friendly menstrual product. Please fill in the answer according to your emphasis on it	
1. Environmental protection characteristics of the product itself.	11. It is convenient to exercise and go into the water.
2. Feel more comfortable physically.	12. Enabling a wider selection of products on the market.
3. There is no excess garbage.	13. Reduce menstrual blood leakage.
4. The overall use cost is cheaper.	14. Worry about cleaning or disinfection trouble.
5. It can be used with other menstrual products.	15. Need to clean by hand, afraid of hands touching menstrual blood.
6. When cleaning, you can conveniently observe whether the menstrual blood is healthy or not.	16. Require cleaning and afraid of not being able to wash it clean.
7. It can be replaced for a long time.	17. The process of learning and getting started is more troublesome.
8. The product is unique.	18. Fear of pain from invasive products.
9. The project is innovative.	19. Fear of invasive products damaging the hymen.
10. No need to carry extra menstrual supplies for replacement.	20. Bacterial infection caused by improper handling of invasive products.

**Table 5:** Analysis of consumers’ use of menstrual products.

		Never Used/Not Using	Used/Using
Menstrual pads	Number of subjects	173	267
	Percentage	39.3%	60.7%
Tampons	Number of subjects	270	170
	Percentage	61.4%	38.6%
Cloth menstrual pads	Number of subjects	301	139
	Percentage	68.4%	31.6%
Menstrual cups	Number of subjects	225	215
	Percentage	51.1%	48.9%
Menstrual underpants	Number of subjects	383	57
	Percentage	87.0%	13.0%

**Table 6:** Table of consumers’ cognition of sustainability.

	Item	Strongly Disagree	Disagree	Fair	Agree	Strongly Agree
1	I attach importance to environmental protection and worry about nature being damaged.	5 1.1%	6 1.4%	46 10.5%	220 50.0%	163 37.0%
2	I give priority to environmental friendliness when buying goods.	2 0.5%	15 3.4%	123 28.0%	213 48.4%	87 19.8%
3	I prepare my own eco-friendly bags, chopsticks, and cups, and do not use disposable products, as possible.	3 0.7%	16 3.6%	88 20.0%	207 47.0%	126 28.6%
4	I am willing to buy eco-friendly products even at a high price.	3 0.7%	20 4.5%	127 28.9%	226 51.4%	64 14.5%
5	I reduce waste, and classify and recycle waste.	2 0.5%	2 0.5%	34 7.7%	217 49.3%	185 42.0%
6	I do not think it is eco-friendly to use menstrual tampons.	8 1.8%	31 7.0%	79 18.0%	178 40.5%	144 32.7%
7	I pay special attention to products with eco-friendly certificates when I shop.	6 1.4%	36 8.2%	187 42.5%	153 34.8%	58 13.2%
8	I learn about eco-friendly products online.	4 0.9%	9 2.0%	83 18.9%	218 49.5%	126 28.6%
9	I value ecological conservation and care about the survival rights of other species.	3 0.7%	4 0.9%	83 18.9%	207 47.0%	143 32.5%
10	I think environmental protection is more important than convenience.	3 0.7%	21 4.8%	118 26.8%	203 46.1%	95 21.6%
11	I support products that are more sustainable and eco-friendly.	1 0.2%	0 0.0%	26 5.9%	223 50.7%	190 43.2%
12	I always pay attention to issues related to environmental protection and sustainability.	2 0.5%	4 0.9%	83 18.9%	220 50.0%	131 29.8%

**Table 7:** Table of consumers’ cognition of menstruation.

	Item	Strongly Disagree	Disagree	Fair	Agree	Strongly Agree
1	I’m afraid of having my hymen broken.	219 49.8%	91 20.7%	97 22.0%	27 6.1%	6 1.4%
2	I think invasive menstrual products will break the hymen.	200 45.5%	104 23.6%	78 17.7%	52 11.8%	6 1.4%
3	I think the deep meaning of menstrual blood is dirtiness and filthiness.	324 73.6%	78 17.7%	25 5.7%	11 2.5%	2 0.5%
4	I think menstruation brings inconvenience in life.	14 3.2%	22 5.0%	78 17.7%	217 49.3%	109 24.8%
5	I do not think I should visit a temple during menstruation.	240 54.5%	85 19.3%	69 15.7%	37 8.4%	9 2.0%
6	I think it is inconvenient to exercise during menstruation.	78 17.7%	99 22.5%	119 27.0%	119 27.0%	25 5.7%
7	I feel ashamed and embarrassed about having menstruation.	266 60.5%	118 26.8%	49 11.1%	6 1.4%	1 0.2%
8	I think it’s shameful for people to see menstrual products, so I pack them in special bags.	171 38.9%	139 31.6%	96 21.8%	30 6.8%	4 0.9%
9	I usually refer to menstruation as “that thing”.	85 19.3%	89 20.2%	109 24.8%	120 27.3%	37 8.4%
10	I think menstruation is a normal physiological phenomenon of women.	0 0.0%	0 0.0%	7 1.6%	99 22.5%	334 75.9%
11	I support gender equality and do not subscribe to the traditional idea that men and women are different.	2 0.5%	2 0.5%	31 7.0%	117 26.6%	288 65.5%
12	I think menstruation is an important cycle of life reproduction and should be valued.	0 0.0%	1 0.2%	32 7.3%	139 31.6%	268 60.9%
13	Women should have the right to take menstrual leave from school or work.	0 0.0%	0 0.0%	23 5.2%	127 28.9%	290 65.9%
14	I tend to hide and keep silent regarding self-care during menstruation.	105 23.9%	152 34.5%	127 28.9%	48 10.9%	8 1.8%
15	I am afraid to touch my private parts directly.	211 48.0%	116 26.4%	82 18.6%	26 5.9%	5 1.1%

**Table 8:** Table of explanatory variations after rotation of the lifestyle scale.

Initial Eigenvalue			
Component	Eigenvalue	Explanatory variation (%)	Accumulative explanatory variation (%)
1	3.654	22.840	22.840
2	1.643	10.269	33.109
3	1.380	8.626	41.735
4	1.199	7.493	49.228

**Table 9:** Component matrix of principal components of the lifestyle scale after analysis rotation.

Item	Factor Component			
	1	2	3	4
Item 3-06	0.684	0.064	0.318	0.016
Item 3-07	0.667	0.079	−0.014	0.099
Item 3-05	0.663	0.042	0.205	−0.191
Item 3-15	0.627	0.141	−0.290	0.114
Item 3-14	0.572	0.239	−0.345	0.052
Item 3-09	0.571	0.308	0.168	−0.108
Item 3-18	0.134	0.756	−0.041	−0.122
Item 3-01	0.019	0.703	0.127	−0.051
Item 3-17	0.180	0.702	0.000	0.098
Item 3-19	0.322	0.569	0.073	0.072
Item 3-20	0.041	0.509	0.147	0.142
Item 3-12	−0.057	0.072	0.771	0.182
Item 3-13	0.190	0.251	0.553	−0.035
Item 3-22	−0.064	0.097	−0.185	0.705
Item 3-21	−0.015	−0.154	0.227	0.703

The background color represents the same set of factors.

**Table 10:** Table of factor constructs and mean coefficient values of different lifestyle clusters.

	Cluster 1 People Seeking New Information and New Trends	Cluster 2 People Subject to Economic Restriction and Wanting to Try Something New	Cluster 3 People Pursuing a Conservative and Stable Life	F-Test	*p*-Value
Factor 1 new information seeking	0.54445	0.50325	−0.78749	152.284	0.000
Factor 2 innovation trend	0.28663	0.59175	−0.70778	115.160	0.000
Factor 3 strict budgeting	−0.46268	0.70748	−0.34917	85.087	0.000
Factor 4 easy-going and conservative	−0.96672	0.62362	0.03712	128.074	0.000
Number of subjects	108	157	175		
Percentage (%)	24.5	35.7	39.8		
Name of cluster	People seeking new information and new trends	People subject to economic restrictions and wanting to try something new	People pursuing a conservative and stable life		

**Table 11:** Table of explanatory variations after rotation of the purchase consideration scale.

Initial Eigenvalue			
Component	Eigenvalue	Explanatory Variation (%)	Accumulative Explanatory Variation (%)
1	5.431	19.395	19.395
2	4.400	15.715	35.109
3	1.911	6.825	41.934
4	1.774	6.337	48.271
5	1.439	5.139	53.410
6	1.182	4.222	57.632
7	1.064	3.798	61.430
8	1.001	3.575	65.006

**Table 12:** Component matrix of principal components of the purchase consideration scale after analysis rotation.

Item	Factor Component							
	1	2	3	4	5	6	7	8
Item 2-18	0.754	−0.255	0.035	0.126	0.050	0.082	−0.041	0.110
Item 2-16	0.738	0.043	0.104	0.020	−0.108	−0.077	0.156	−0.068
Item 2-14	0.719	0.137	0.035	0.132	−0.146	−0.152	0.085	−0.125
Item 2-20	0.717	−0.043	0.131	0.033	−0.091	0.040	0.080	0.232
Item 2-15	0.688	−0.067	−0.118	0.212	0.078	−0.066	0.004	−0.299
Item 2-17	0.646	−0.011	0.284	−0.013	0.114	−0.091	−0.050	0.000
Item 2-19	0.617	−0.274	−0.164	0.147	0.197	0.067	0.071	−0.051
Item 2-10	−0.005	0.761	0.061	−0.009	0.193	0.195	−0.017	0.128
Item 2-11	−0.082	0.743	0.098	0.041	0.205	0.091	0.042	0.061
Item 2-13	−0.028	0.722	0.049	0.136	−0.120	0.047	0.140	0.053
Item 2-07	−0.184	0.695	0.013	−0.088	0.242	0.224	−0.093	−0.021
Item 1-02	0.124	−0.068	0.788	0.061	0.179	0.104	−0.052	0.013
Item 1-03	0.051	0.033	0.782	0.006	0.080	0.137	−0.005	0.191
Item 1-04	0.072	0.209	0.652	0.088	0.014	0.051	0.194	0.190
Item 1-01	0.053	0.168	0.548	0.231	−0.111	0.228	0.163	−0.232
Item 1-06	0.217	0.008	0.049	0.861	0.094	−0.038	0.031	0.046
Item 1-07	0.149	−0.033	0.088	0.859	0.121	−0.005	0.025	0.125
Item 1-08	0.087	0.163	0.119	0.609	0.140	0.184	0.295	−0.124
Item 2-08	−0.004	0.228	0.106	0.158	0.848	0.140	0.160	0.052
Item 2-09	0.016	0.215	0.121	0.234	0.823	0.137	0.156	0.049
Item 2-04	−0.014	0.213	0.127	0.094	0.058	0.745	−0.117	0.143
Item 2-05	−0.083	0.198	0.255	−0.034	0.011	0.712	0.236	0.027
Item 2-06	−0.093	0.140	0.077	−0.001	0.297	0.642	0.218	0.150
Item 1-10	0.245	−0.011	0.075	0.175	0.124	−0.007	0.723	−0.092
Item 1-09	0.019	0.067	0.064	0.077	0.147	0.237	0.715	0.250
Item 2-02	−0.048	0.332	0.133	0.080	−0.053	0.167	−0.154	0.643
Item 2-01	−0.078	−0.033	0.084	−0.018	0.132	0.272	0.376	0.640

The background color represents the same set of factors.
